# Modelling pathogen spread in a healthcare network: Indirect patient movements

**DOI:** 10.1371/journal.pcbi.1008442

**Published:** 2020-11-30

**Authors:** Monika J. Piotrowska, Konrad Sakowski, André Karch, Hannan Tahir, Johannes Horn, Mirjam E. Kretzschmar, Rafael T. Mikolajczyk

**Affiliations:** 1 Institute of Applied Mathematics and Mechanics, University of Warsaw, Warsaw, Poland; 2 Institute of High Pressure Physics, Polish Academy of Sciences, Warsaw, Poland; 3 Research Institute for Applied Mechanics, Kyushu University, Kasuga, Fukuoka, Japan; 4 Institute for Epidemiology and Social Medicine, Münster, Germany; 5 Julius Center for Health Sciences & Primary Care, University Medical Center Utrecht, Utrecht University, Utrecht, The Netherlands; 6 Institute for Medical Epidemiology, Biometrics and Informatics, Martin Luther University Halle-Wittenberg, Halle (Saale), Germany; University of California Irvine, UNITED STATES

## Abstract

Inter-hospital patient transfers (direct transfers) between healthcare facilities have been shown to contribute to the spread of pathogens in a healthcare network. However, the impact of indirect transfers (patients re-admitted from the community to the same or different hospital) is not well studied. This work aims to study the contribution of indirect transfers to the spread of pathogens in a healthcare network. To address this aim, a hybrid network–deterministic model to simulate the spread of multiresistant pathogens in a healthcare system was developed for the region of Lower Saxony (Germany). The model accounts for both, direct and indirect transfers of patients. Intra-hospital pathogen transmission is governed by a SIS model expressed by a system of ordinary differential equations. Our results show that the proposed model reproduces the basic properties of healthcare-associated pathogen spread. They also show the importance of indirect transfers: restricting the pathogen spread to direct transfers only leads to 4.2% system wide prevalence. However, adding indirect transfers leads to an increase in the overall prevalence by a factor of 4 (18%). In addition, we demonstrated that the final prevalence in the individual healthcare facilities depends on average length of stay in a way described by a non-linear concave function. Moreover, we demonstrate that the network parameters of the model may be derived from administrative admission/discharge records. In particular, they are sufficient to obtain inter-hospital transfer probabilities, and to express the patients’ transfers as a Markov process. Using the proposed model, we show that indirect transfers of patients are equally or even more important as direct transfers for the spread of pathogens in a healthcare network.

## Author summary

Direct patient transfers between hospitals have been shown to play an important role in the spread of pathogens in a healthcare network. However, readmission of patients from the community (indirect transfers) to the same or a different hospital is not well studied, and its role for the spread of pathogens in a healthcare network is not quantified. In this work, we developed a network model of a healthcare system to study the impact of indirect transfers on the prevalence in the individual hospitals as well as in the overall healthcare system. The model includes both, direct and indirect transfers of patients between the healthcare facilities due to transferring as well as readmission of infectious (colonized or infected) patients. Our results show that the readmission of patients (indirect transfers), either to the same or different facility, is an important potential channel of pathogen transmission. Such indirect transfers are of no less importance than direct patient transfers in controlling the spread of pathogens in a healthcare network.

## Introduction

Recent years brought an increased attention to the question of how patient traffic between healthcare facilities contributes to the spread of healthcare-associated infections in general, and of multidrug resistant pathogens in particular [1]. The movements of patients between hospitals can be divided into transfers of patients from one hospital to another (i.e. direct transfers), and in readmission of patients to the same or another hospital after having spent some time in the community (i.e. indirect transfers). Based on patient movements, one can derive a healthcare network, where nodes represent hospitals and communities, whereas connections between the nodes reflect patient transfers. Both, direct and indirect transfers may contribute to the spread of pathogens in healthcare networks. Effects of interventions to prevent the spread of pathogens in such healthcare networks may differ for direct and indirect transfers. While screening of patients, who are transferred from one hospital to another is an obvious intervention measure, this is less clear for patients who are readmitted from the community [2]. The effectiveness/cost-effectiveness of screening of patients after an indirect transfer depends on the time between admissions, the clearance rate of the pathogen, and whether screening is applied in a targeted way, i.e. based on individual risk factors of the patient. To quantify the effectiveness of such measures, we need to understand the contribution of indirect transfers to the spread of pathogens through the network. In the past decade, eleven studies assessed healthcare networks in countries or federal states. All of these used national or federal registries in the US [3–7] or Europe (England, France, Germany, the Netherlands) [1, 8–12]. The definition of transfer and patient movement was quite heterogeneous. While some studies took into account direct and indirect transfers independently of the length of the community stay between two hospital admissions [8, 10], others restricted indirect transfers to readmissions within 90 [7] or 365 days [12], or did not consider them at all [6] when deriving hospital network flow characteristics. For studies which included indirect transfers, sometimes the description of how such transfers were incorporated is not clear. Moreover, only preliminary analyses were conducted for Germany [10]. Such heterogeneities make it difficult to compare the findings. In this work, we aim to develop a model to explicitly account for indirect transfers using data from one regional healthcare insurance company in Germany and to study the impact of such transfers on the spread of multiresistant pathogens in a healthcare network. We use methicillin-resistant *Staphylococcus aureus* (MRSA) as an example pathogen and describe selected implications of either including or ignoring indirect transfers for the spread of a MRSA strain transmitted mainly in the hospitals.

## Materials and methods

### Description of dataset

In the German healthcare system, over 90% of the population is insured by public insurance companies and the remaining population by private insurance companies. Overall, there exist more than 190 public insurance companies. These scattered data together with high standards of data protection are a reason that reimbursement data in Germany are less accessible for scientific analysis than in other countries. We used an anonymized hospital discharge dataset provided by AOK Lower Saxony, a statutory regional healthcare insurance company. AOK Lower Saxony includes almost exclusively residents of the federal state of Lower Saxony and covers around 30% of the local population in this federal state.

The dataset contains hospitalisation records of 1 673 247 patients for the years 2008 to 2015. For each hospital stay, the anonymized patient ID, the anonymized healthcare facility ID, the federal state where the healthcare facility is located, day of admission, day of discharge, discharge diagnosis (ICD 10 GM code), as well as age and sex of the patient are available. For data protection reasons, exact geographical locations of the healthcare facilities were not provided. In the dataset, we identified 4 573 584 hospitalisations in 223 healthcare facilities located in Lower Saxony, and 680 908 hospitalisations in healthcare facilities in other German federal states.

We excluded a subset of facilities because either they had very few hospitalisations in the eight-year period, or they were not operational (no hospitalisations) for a sufficient time interval. Thus, we finally considered 164 healthcare facilities located in Lower Saxony.

For more detailed information on e.g. sex of the patients, distributions of length of hospital stays, detailed characteristics of overlaps as well as a technical description of the provided dataset, we refer to a technical report [13].

### Model input data

**Fig 1:**
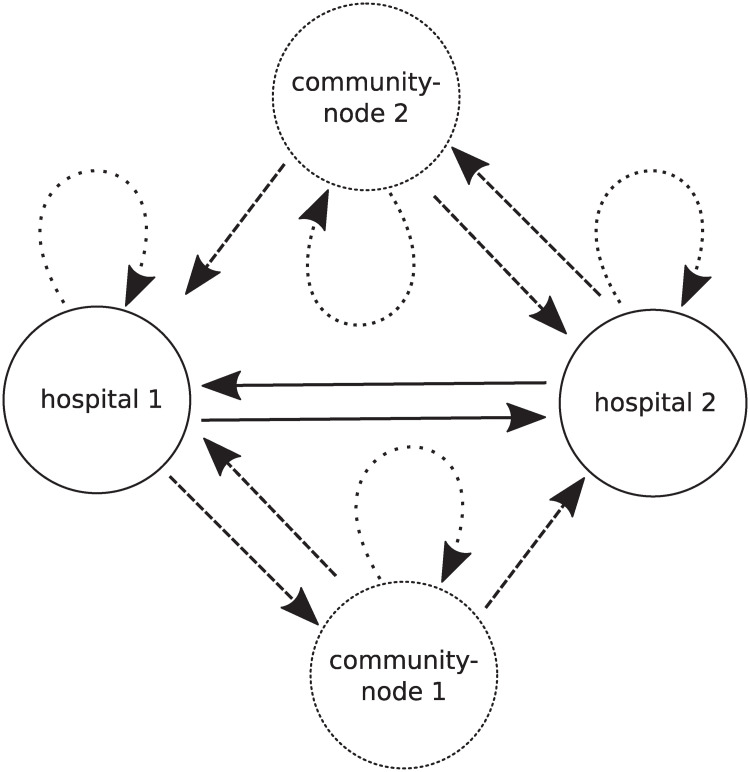
Graphical representation of transfers in a model comprising two exemplary hospitals. Direct transfers are represented by solid lines, while indirect transfers are represented by dashed lines. Dotted loops indicate the situations when patients stay for a night in the hospitals/community nodes.

We constructed a *directed*, *weighted graph*, based on patient discharge data. In such a network, *nodes* represent healthcare facilities and *weights* correspond to direct transfer probabilities. To account for indirect transfers, we define additional *community-nodes* (see Sec. Sizes of community-nodes), which correspond to the communities associated with the healthcare facilities. Patients leaving a healthcare facility go to such a community-node, and then they may be readmitted to the same or another facility, c.f. Fig 1. As a result, the model allows to simulate the patient transfers from healthcare facilities to communities and back to healthcare facilities.

#### Extraction of direct and indirect transfers from data

We distinguish two types of transfers. A *direct transfer* is when patients move from one facility to the next on the same or the next day. In contrast, an *indirect transfer* means a new admission to the same or a different hospital after at least one day outside of any hospital. We define duration of a transfer as the time between discharge and the next admission. The situation when the patient is re-admitted to the same facility after some time spent in the community is called *(indirect) auto-transfer*.

**Fig 2:**
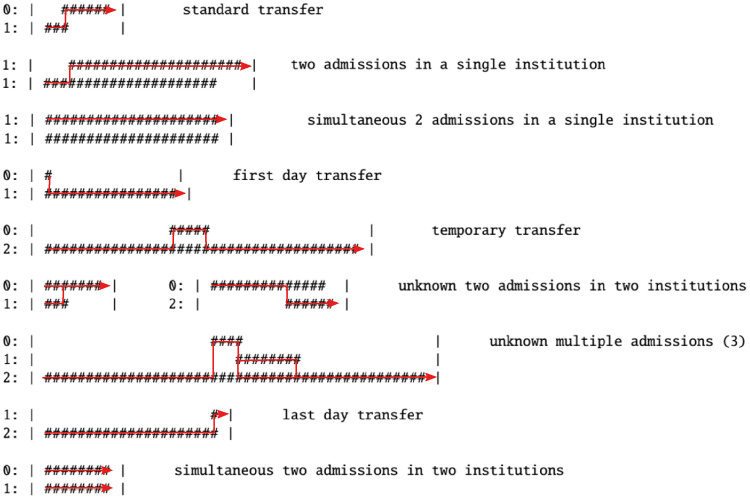
Examples of the results of the transfer detection algorithm in case of different types of overlaps. Results are represented by red lines, numbers on the left-hand side indicate the numeric codes for particular units, while # stand for days of stay reported for a given healthcare facility. For the classification of particular types of overlaps see [13].

The direct transfers can be subject to overlapping (one day or longer) hospital stays, i.e. for the same patient at least two hospitalisations are reported with overlapping periods (c.f. Fig 2). We detected 304 833 overlapping cases for healthcare facilities located in Lower Saxony only. Several factors contribute to the existence of overlaps. First, the granularity of admission/discharge is in days, causing that a transfer may be indicated by a single-day overlap (112 368 cases corresponding to 38.1% of all detected overlaps) as well as two consecutive non-overlapping stays. Moreover, an overlap may be longer than one day if a patient returns to the originating facility and his/her stay continues. Overlaps may also occur due to coding errors in the original dataset which cannot be corrected given the anonymized nature of the data. For further details on overlaps, we refer the reader to an open access report [13].

To deal with overlaps of more than one day, we derived an algorithm declaring in which hospital is the patient on the given day (see S2 Appendix for details). Using this algorithm, we identified 2 733 286 transfers in Lower Saxony, among which 157 143 were direct transfers (below 6% of all transfers) and 2 576 143 were indirect transfers (above 94%, including 1 648 400 auto-transfers).

**Fig 3:**
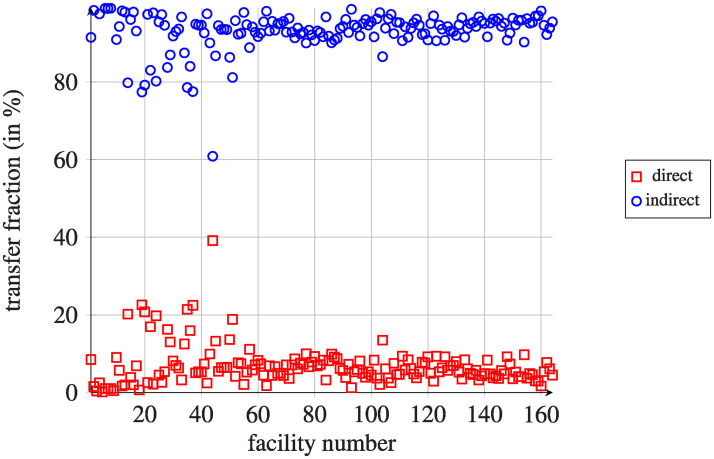
Fractions of outgoing direct and indirect transfers per day. Results are presented for all considered healthcare facilities (164 units), which are ordered by the mean size of the units, the smallest first.

Fig 3 shows the fractions of both detected types of transfers for each facility. For indirect transfers, the average duration of stay in the community for indirect transfers was 320.1 days (SD = 435.2), while the average length of stays in hospitals was 8.7 days (SD = 12.2). There were only 573 045 indirect transfers (about 20% of all transfers) with a community stay duration of less than 30 days.

#### Sizes of hospital nodes

**Fig 4:**
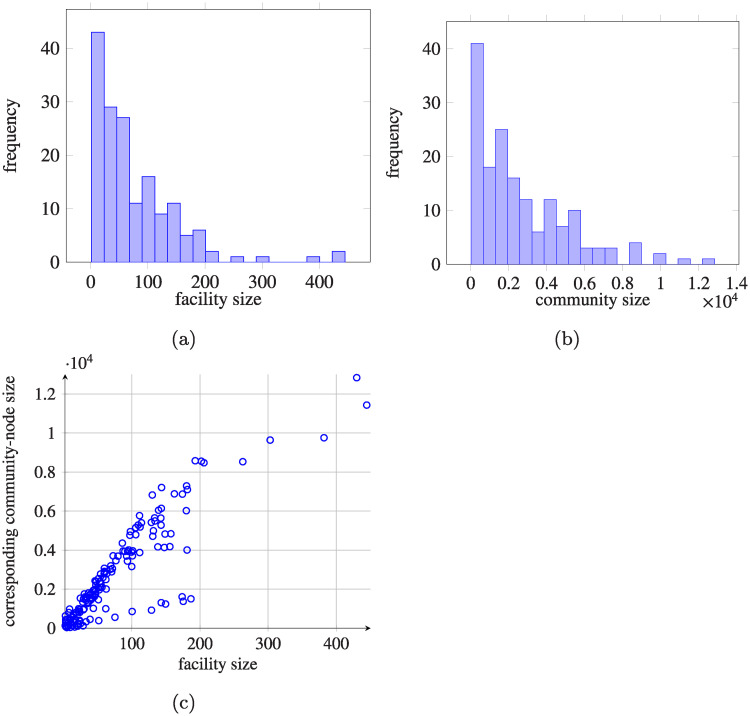
Hospital and community sizes analysis. (a) Histogram of estimated hospital sizes, defined as average numbers of patients in the hospitals within the period 2008–2015, calculated for each healthcare facility separately (164 units)—given the fact that AOK Lower Saxony covers 30.52% of the population in Lower Saxony, the number of beds has to be multiplied by about 3 to get the actual sizes; (b) histogram of estimated community sizes, defined as average numbers of patients in the given community within the period 2008-2015, calculated for each healthcare facility separately, as above the populations have to be multiplied; (c) dependence of community-node sizes on corresponding healthcare facilities (164 units).

Since healthcare facilities are anonymized in the dataset, we do not have information on their sizes. To estimate them (for 164 units), we counted all patients present on a given day in a given facility, and then took the average over the facility operation period. The distribution of the resulting hospital sizes is presented in Fig 4. In addition, in S4 Appendix some examples of the length of stay histograms for considered facilities are presented.

#### Sizes of community-nodes

To estimate the average number of people being subject to indirect transfers, we first investigated the change in time of the number of people being in community between two hospitalisations. To keep track of the originating hospital of a community dwelling patient, we created a *community-node* for each facility *i*, indexed by *n* + *i*, where *n* is the number of all considered healthcare facilities. A patient was assumed to be in the community-node *n* + *i* if the patient was discharged from the healthcare facility *i* and after some time was admitted to another (or possibly the same) healthcare facility *j* ∈ {1, …, *n*} or was not admitted to any hospital in the considered period.

To estimate the average sizes of community-nodes, we iterated through the dataset period and we calculated the number of patients being in indirect transfer, discharged from a given facility, and waiting for their admission to another one for every day and for every facility. For *i* community node we also counted patients who were subject to first hospital stay in unit *i*. Then we took the means of these numbers. Fig 4 shows the relationship between healthcare facility sizes and sizes of the corresponding community-nodes i.e. persons discharged from healthcare facilities and waiting for (re)admission.

### Model description

We describe the healthcare system as a graph, where the nodes represent healthcare facilities and corresponding community-nodes, with weighted edges representing the probabilities of transfers (both direct and indirect) between the nodes.

#### Patient traffic

To construct the healthcare network, the model requires as an input a list of healthcare facilities (*n* elements) and corresponding community-nodes (*n* elements) with a matrix $A={\left[A_{ij}\right]}_{i,j=1}^{2n}$ determining the transfer probabilities between the nodes.

Hospital discharge data were collected for a period of D∈N days, thus by D≔{1,…,D} we denote the set of all days for which admissions and discharges are registered. This means that every number in D set corresponds to a single reported day.

Let *t*_*ij*_(*d*), $t_{ij}:D\to N_{0}$ for any *i* ≠ *j* denote a number of transitions from node *i* to node *j*, within a single day d∈D, which can be derived from the dataset provided by the insurance company (see S2 Appendix).

We differentiate four categories of transfers, depending on indices *i*, *j*:

- *i*, *j* ∈ {1, …, *n*}—direct transfers between healthcare facilities;
- *i* ∈ {*n* + 1, …, 2*n*}, *j* ∈ {1, …, *n*}—indirect transfers from community-nodes to healthcare facilities;
- *i* ∈ {1, …, *n*}, *j* ∈ {*n* + 1, …, 2*n*}—transfers from healthcare facilities to community-nodes. These may also be interpreted as initiations of indirect transfers. Since a patient stays in the community-node corresponding to the discharging facility, only *t*_*i*,*n*+*i*_ ≠ 0, and for *j* ≠ *n* + *i* we have *t*_*ij*_ ≡ 0;
- *i*, *j* ∈ {*n* + 1, …, 2*n*}—transfers between community-nodes. Due to the nature of community-nodes, there is no transfer between them, thus these elements are zero except for *t*_*ii*_.

Let $T={\left[T_{ij}\right]}_{i,j=1}^{2n}$ be a matrix of aggregated numbers of all direct and indirect transfers between nodes within a given time period:

$$\begin{array}{c} T_{ij}=\sum_{d=1}^{D}t_{ij}\left(d\right). \end{array}$$ (1)

We refer to each node (hospital or community-node) by their index in the matrix *T*.

Now, we define *a*_*ij*_(*d*) to be a transfer probability per patient from node *i* to node *j* in a single given day d∈D. Then for an arbitrary chosen facility, the average number of transferred patients at a given day is expressed by *a*_*ij*_(*d*)*p*_*i*_, where *p*_*i*_ is the average population size in the *i*-th node, to be defined later. Thus,

$$\begin{array}{c} t_{ij}\left(d\right)\equiv a_{ij}\left(d\right)p_{i}. \end{array}$$ (2)

Assuming that the probability of a transfer from node *i* to node *j* in a single given day does not depend on the choice of a day, i.e. *a*_*ij*_(*d*) ≡ *A*_*ij*_ = const, we get

$$\begin{array}{c} T_{ij}=\sum_{d=1}^{D}t_{ij}\left(d\right)=DA_{ij}p_{i}, \end{array}$$ (3)

and we determine non-diagonal elements of the matrix *A* as

$$\begin{array}{c} A_{ij}=\frac{T_{ij}}{Dp_{i}}=\frac{1}{Dp_{i}}\sum_{d=1}^{D}t_{ij}\left(d\right). \end{array}$$ (4)

With elements *A*_*ij*_ ≔ *A*_*ij*_(*p*_*i*_) defined, we may easily determine the diagonal elements *A*_*ii*_ ≔ *A*_*ii*_(*p*_*i*_), corresponding to the probability of staying in a given node as

$$\begin{array}{c} A_{ii}≔1-\sum_{j\neq i}A_{ij}, \end{array}$$ (5)

and thus matrix *A* of a Markov chain is determined up to node average populations **p** = [*p*_1_, …, *p*_2*n*_] and 0 ≤ *A*_*ii*_ ≤ 1. We picked the values of *p*_*i*_ such that the average length of stay of the patients in the nodes agree with the values determined directly from the data.

By the success at a given day *k*, let us denote a situation when a given patient leaves the hospital. It means that for *k* − 1 days, with probability of *A*_*ii*_ for each day, the patient was in the hospital and then, at day *k*, the patient was dismissed with probability 1 − *A*_*ii*_. Thus, we get $A_{ii}^{k-1}\left(1-A_{ii}\right)$ that is the probability mass function of the geometric distribution. Therefore, average patient length of stay in a given hospital *i* can be estimated using 1/(1 − *A*_*ii*_).

**Fig 5:**
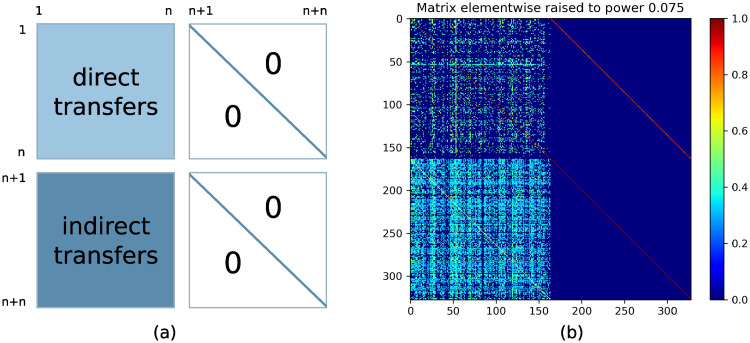
Visualization of the transfer probability matrix. Healthcare facilities are numbered from 1 to *n* and community-nodes numbered from *n* + 1 to *n* + *n*, *n* = 164. (a) schema presenting four distinguishable blocks; (b) quantitative representation of obtained probabilities, colour pixels denote the probabilities; for the purpose of visualization, all elements were raised to the power 0.075.

The transfer probability matrix obtained by the above procedure is visualized in Fig 5. It has a block structure with the indirect transfer block clearly denser (see discussion in Section Extraction of direct and indirect transfers from data). The upper right diagonal block corresponds to discharge from healthcare facilities to community-nodes, and thus it accounts for total indirect transfer quantity. The lower right diagonal block corresponds to patient exchange between community-nodes. Since community-nodes are assumed to be separated, only the diagonal elements are present to account for patients remaining at the community-nodes.

In our model, patient traffic is simulated as follows. Assume that $q^{0}≔\left[q_{1}^{0},\ldots ,q_{2n}^{0}\right]$ is an initial patient probability distribution in healthcare facilities and community-nodes (a vector of the number of patients in each node, normalised to a sum of 1). The probability changes in the following days is calculated by iterating

$$\begin{array}{c} q^{k}≔q^{k-1}A=q^{0}A^{k}. \end{array}$$ (6)

If we start with **q**^0^ being an eigenvector of *A* (i.e. **q**^0^*A* = λ**q**^0^, where λ is an eigenvalue of matrix *A*), we get **q**^*k*^ = λ^*k*^**q**^0^. Moreover, if matrix *A* represents a *regular* Markov chain (i.e. there exists *k* such that all elements of *A*^*k*^ are positive), then we can find the stationary probability distribution **w** of the patients in hospitals for our network model by solving system **w***A* = **w**. In such a case λ = 1 is an eigenvalue of the matrix *A*. On the other hand, if the considered Markov chain is *absorbing*, then we would be able to calculate the probability of absorption from one state to another and the average number of steps before the absorption happens. It is natural to expect that a Markov chain describing the patient transfer process is not absorbing, as no such phenomenon is observed in real healthcare systems.

**Fig 6:**
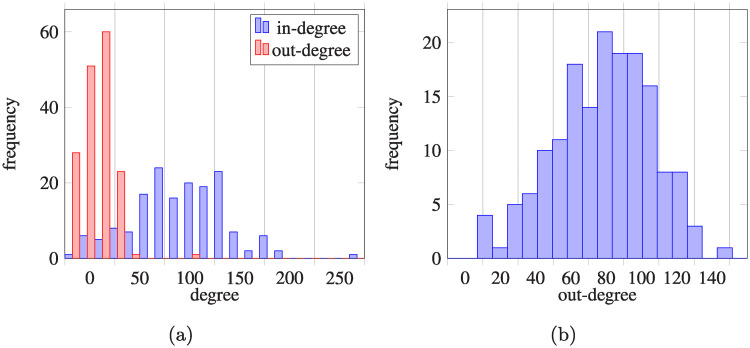
Degrees of the nodes in the hospital network. (a) In-degree and out-degree for each hospital node of the network (self-loops not included); (b) out-degree for each community node of the network (self-loops not included), in-degree for all community nodes are equal to 1.

In our analysis, we mostly discuss the stationary distribution of patients counted as a number of patients in a given healthcare facility, while in the Markov processes we consider the stationary probability distribution i.e. vector of the probabilities of being in a given facility. However, we can easily interchange these vectors by dividing/multiplying them by the number of patients in the healthcare system. In case of the network derived from the provided data, the regularity of matrix *A* was checked numerically by empirical verification (*A*^5^ has positive elements only), but this result is dependent on numerical errors. Nevertheless, we can use a lemma providing an analytical argument, for details see [14] or S1 Appendix. A non-zero pattern of matrix *A* is presented in Fig 5b. Fig 6 shows in- and out-degree of all network nodes, calculated using transfer probability matrix *A*.

#### Pathogen transmission dynamics

To model the spread of MRSA within nodes, we use a *susceptible-infectious-susceptible model* (SIS), [15] expressed by a system of ordinary differential equations. In this classic approach, we assume to have two well mixed and constant in time populations of *P* individuals that can be susceptible or infectious. Infectious individuals include those colonized as well as those who develop MRSA infection and we do not distinguish between these populations. Let *S*_*f*_(*t*) and *I*_*f*_(*t*) denote the fraction of susceptible and infectious individuals at time *t*, respectively. The term *βS*_*f*_(*t*)*I*_*f*_(*t*) describes the infection process due to the contact of susceptible and infectious individuals, while *γI*_*f*_(*t*) describes the recovery process. The equations of the SIS model are:

$$\begin{array}{c} \begin{array}{cc} \frac{d}{dt}S_{f}\left(t\right) & =-\beta S_{f}\left(t\right)I_{f}\left(t\right)+\gamma I_{f}\left(t\right),\\ \frac{d}{dt}I_{f}\left(t\right) & =\beta S_{f}\left(t\right)I_{f}\left(t\right)-\gamma I_{f}\left(t\right), \end{array} \end{array}$$ (7)

where *S*_*f*_ + *I*_*f*_ = 1 due to the definition of *S*_*f*_ and *I*_*f*_, and as a consequence

$$\begin{array}{c} \frac{d}{dt}I_{f}\left(t\right)=-\beta {\left(I_{f}\left(t\right)\right)}^{2}+\left(\beta -\gamma \right)I_{f}\left(t\right), \end{array}$$ (8)

which is a logistic equation, [16], with analytical solutions which exist globally and are unique. Eq 8 has two steady states: a trivial one (locally stable for *β* < *γ* and unstable for *β* > *γ*) and a positive one providing *β* > *γ* (which is stable whenever it exists). Thus, for *β* ≤ *γ* the fraction of infectious individuals goes to zero, while for *β* > *γ* the fraction of infectious individuals stabilizes at a certain level.

In our work, the use of absolute numbers of susceptible and infectious patients *S*, *I* is favourable, therefore we consider

$$\begin{array}{c} S\left(t\right)=S_{f}\left(t\right)\cdot P,\;I\left(t\right)=I_{f}\left(t\right)\cdot P, \end{array}$$ (9)

where *I*_*f*_(*t*) and *S*_*f*_(*t*) are solutions to (7) and *P* denotes the population size in the node.

We apply this simple SIS model to all healthcare facilities and community-nodes in our network. Since we focus only on hospital-acquired infections in this work, we assume no transmission (*β* = 0) in the community-nodes. As a consequence in the following, we assume that **p**^*k*^ = **s**^*k*^ + **i**^*k*^, $s^{k}≔\left[s_{1}^{k},\ldots ,s_{2n}^{k}\right]$, $i^{k}≔\left[i_{1}^{k},\ldots ,i_{2n}^{k}\right]$, where as before $p^{k}=\left[p_{1}^{k},...,p_{2n}^{k}\right]$ denotes the average distribution of the patients in network nodes after *k* iterations, while **i**^*k*^ and **s**^*k*^ stand for average distributions of infectious and susceptible patients in nodes, respectively.

Let us define the numerical subroutine sisolve as follows. For a given *S*_0_, *I*_0_, *t*_0_, *t*_1_, *β*, *γ*, it returns values *S*_1_, *I*_1_ (i.e. the numbers of susceptible and infectious individuals after time *t*_1_ − *t*_0_ = 1 day, respectively), where

$$\begin{array}{c} S_{1}≔S\left(t_{1}\right),\;I_{1}≔I\left(t_{1}\right), \end{array}$$ (10)

where functions *S*, *I* are the solutions of the SIS model (with initial condition *S*(*t*_0_) = *S*_0_ and *I*(*t*_0_) = *I*_0_) given by (9). So basically $\text{sisolve}:R^{6}\to R^{2}$ together with the SIS model defines our modelling approach as follows:

**Require:** $s^{0}=\left[s_{1}^{0},\ldots ,s_{2n}^{0}\right]$, $i^{0}≔\left[i_{1}^{0},\ldots ,i_{2n}^{0}\right]$, T∈N;

1: *t* ≔ 0;

2: **s** = **s**^0^;

3: **i** = **i**^0^;

4: **while** *t* < *T* **do**

5:  **for all** *j* ∈ {1, …, 2*n*} **do**

6:   (*s*_*j*_, *i*_*j*_) ≔ sisolve(*s*_*j*_, *i*_*j*_, *t*, *t* + 1, *β*, *γ*);

7:  **end for**

8:  **s** ≔ **s***A*;

9:  **i** ≔ **i***A*;

10:  **s**^*t*^ ≔ **s**; **i**^*t*^ ≔ **i**;         ⊳ These are the results

11: **end while**

Loop 5 can be parallelized. This is also true for multiplications 8, 9, but here some inter-process information exchange is necessary. Moreover, for our designed numerical procedure, there is no need of having the same recovery and transmission rates for all healthcare facilities and/or community-nodes. The results of the described algorithm, i.e. distribution of susceptible and infectious patients versus discrete time steps, are computed in line 10.

We assume that the time-step is one day, and that the recovery and transmission rates do not vary between the facilities. This behaviour may be easily modified if necessary since the developed code allows to run the simulations with non-homogenous parameters. However, since there is no information available how recovery or transmission rates might differ between hospitals (unlike within), we did not implement this in the primary analysis. We also assume that the transfer probabilities for susceptible and infectious individuals are constant in time (lines 8, 9). To change this assumption, we would have to generate different Markov Chain matrices for these groups, but otherwise the presented algorithm remains unchanged.

### Estimation of SIS parameters

Model parameters for MRSA were based on reference values from the literature. Where necessary, we varied the reference values to study the behaviour of our model under a borader set of assumptions. Parameter values and applied ranges are shown in Table 1. The recovery rate *γ* is equal to one over the mean time spent by an individual in the infectious state and it is measured in units of day^−1^. Following Scanvic et al. [17] and Donker et al. [8, 18], we assumed that the mean time of MRSA colonization is 365 days leading to *γ* = 1/365 day^−1^. In addition, we assumed that recovery rates in particular facilities do not depend on patient characteristics or healthcare facility characteristics itself. Thus, we used the same value of recovery rate for all healthcare facilities and community-nodes, if not indicated otherwise. MRSA transmission rates have been parametrized heterogeneously in previous modelling studies [18, 19], and were often not reported explicitely [8, 9]. We used a value compatible with literature values for the transmission rate within hospitals in our base case scenario, and varied it for further analyses. As stated earlier, we do not consider transmission in the community-nodes; therefore, we set transmission rates for community-nodes to zero. The rationale for this was that we were specifically interested in hospital-associated infections and the role of indirect transfers for their transmission. For the example of MRSA, this would correspond to healthcare-associated MRSA strains (HA-MRSA) which are associated with only few transmissions in the community [20]. While in general some transmission in community may exist, with much smaller transmission parameter, it would not substantially affect the result of simulation (c.f. S3 Appendix). For the set of simulations presenting the impact of the SIS model parameters on the dynamics of the whole model, we varied both SIS parameters recovery rate and transmission rate, as reported in Table 1 (set type: parameter analysis).

**Table 1:** SIS parameters used in simulations.

Set type	Parameter	Reference values	Unit	Reference
MRSA	Recovery rate (*γ*)	1/365	day^−1^	[8, 17, 18]
	Transmission rate in hospital (*β*)	0.06	day^−1^	see Section Estimation of SIS parameters
	Transmission rate in community (*β*)	0	day^−1^	assumed
Parameter analysis	Recovery rate (*γ*)	0.5/365–8/365	day^−1^	assumed
	Transmission rate in hospital (*β*)	0.04–0.85	day^−1^	assumed
	Transmission rate in community (*β*)	0	day^−1^	assumed
Indirect transfer impact	Recovery rate in hospital (*γ*_*h*_)	1/365	day^−1^	[8, 17, 18]
	Recovery rate in community (*γ*_*c*_)	0.125/365–4096/365	day^−1^	see Section Estimation of SIS parameters
	Transmission rate in hospital (*β*)	0.06	day^−1^	see Section Estimation of SIS parameters
	Transmission rate in community (*β*)	0	day^−1^	assumed

For the set of simulations investigating the impact of indirect transfers, we started with a specific parameter combination, *γ*_*h*_ ≔ *γ*_*c*_ = 1/365 day^−1^ and *β* = 0.06 day^−1^, resulting in stabilization of the system-wide community prevalence within a period of 7000 days at the level of 6.7%, and stabilization of the system-wide hospital prevalence at the level of 17.8%. The latter prevalence was close to the mean prevalence reported in [8, 9, 18].

In subsequent simulations, we modelled the effect of increased indirect transfers on pathogen transmission between the facilities by varying the *γ*_*c*_ parameter. We started with the value 0.125/365 day^−1^ ≈ 3.42 × 10^−4^ day^−1^ corresponding to much lower (than the one proposed in [8, 17, 18]) recovery in community. Then, we gradually increased the recovery rate in the community up to 4096/365 day^−1^ ≈ 11 day^−1^, which gives a mean duration of recovery of about two hours. Using this approach, the movement of patients in the system remains the same, but the patients recover faster in the community, so that pathogen transmission through this channel is reduced, and in the most extreme case (*γ*_*c*_ = 4096/365 day^−1^ ≈ 11 day^−1^) it is virtually disabled. In this case, the transmission is limited to the hospital environment only.

### Numerical simulation setup

For simulations of the pathogen spread in the healthcare network, we used software developed by K. Sakowski and M.J. Piotrowska (for details and documentations see [21]). The software is written in Python and comprises two main modules: one for patient transfers between healthcare facilities and one for pathogen spread within healthcare facilities. The former module is superior to the latter one in the sense that it governs the simulation flow. Moreover, it performs the parallelization through the MPI library.

In the first step, the healthcare facility nodes and their community-nodes are distributed between the available processors. Next, the transfer matrix, described in detail in Section Patient traffic, is divided into submatrices, corresponding to blocks of nodes given to the subsequent processes; then these blocks are also distributed to the corresponding processors. The internal model, responsible for calling independent instances of the intra-facility model for pathogen spread simulation with a SIS model as defined in Section Pathogen transmission dynamics, is then initialized. After the intra-facility simulations (covering a one day period) are finished, the patients are transferred between nodes and this process is repeated for the next day, and so on.

### Simulation plan

Using the static network structure described above and the SIS transmission model, we first assess how MRSA spreads in the network including direct and indirect transfers. We further investigate the effects of parameter values on the spread of MRSA within healthcare facilities and corresponding communities. We also check if the starting point of MRSA spread strongly influences model dynamics.

A particular focus is placed on the role of transmission parameters and the initial infection point for the behaviour of the system until a steady state is reached, and on the prevalence of MRSA colonization during steady state in individual healthcare facilities and their corresponding community nodes. By increasing the recovery rate in community-nodes from 0.125/365 day^−1^ to about 4096/365 day^−1^ (instant recovery in the community), we investigate the effects of stepwise restricting the contribution of indirect transfers to the spread of the pathogen.

## Results

### Impact of the initial infection point

Independently of the initial starting point of pathogen spread, we observed three phases of MRSA spread in our network. In the initial phase (time to reach the prevalence of 10% of the final prevalence, see also S5 Appendix), the overall prevalence is small, close to zero. With time, prevalence increases slowly mainly in the initially colonized facility. Then, there is a transition phase (time from the end of phase 1 till reaching the level of 99.9% of final prevalence), where the number of infectious patients increases rapidly. Then, the final phase is reached in individual facilities where the proportion of infectious individuals does not change substantially any more.

**Fig 7:**
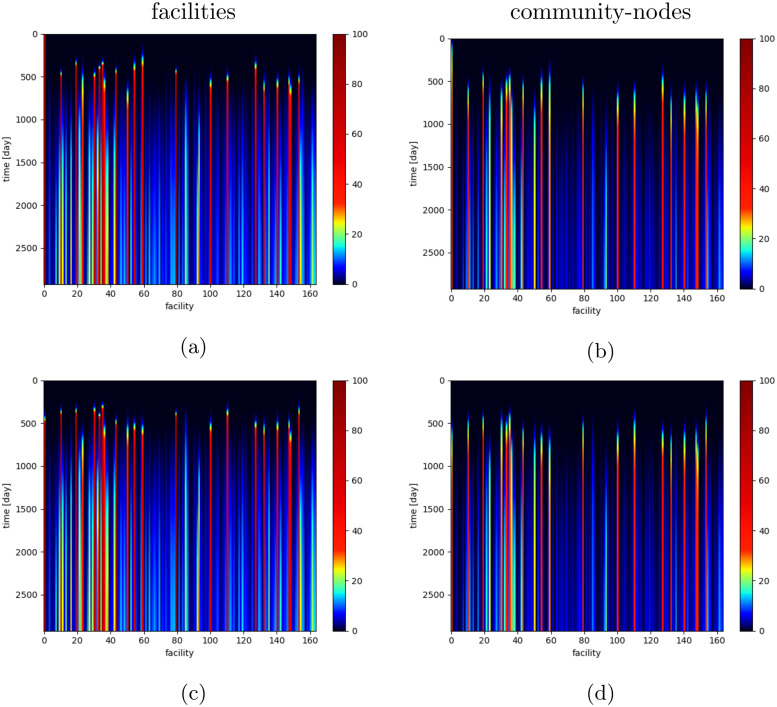
MRSA prevalence in the individual healthcare facilities and community-nodes. MRSA prevalence (over time) expressed as the percentage of infectious individuals per healthcare facility (a,c) and corresponding community-node (b,d). Deep blue corresponds to low infection proportions (lower than 5%); yellow or red to facilities with high infection proportions (higher than 20%). Healthcare facilities are ordered by average size, with the smallest first. The process was started by a single infectious patient located in facility number: (a,b) 1 (the smallest facility); (c,d) 164 (the biggest facility).

**Fig 8:**
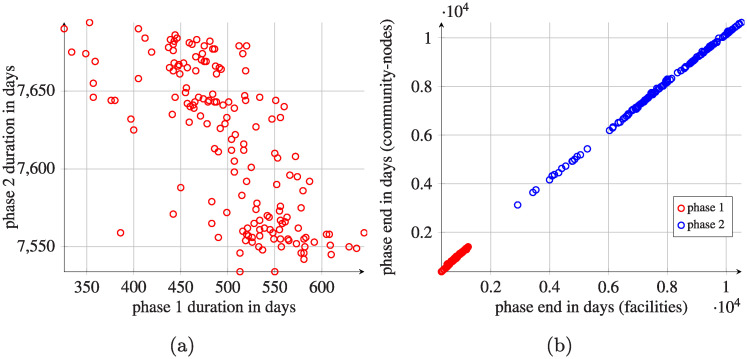
Comparison of average system-wide phase durations and individual phase ends expressed in days in simulations when each healthcare facility was selected as the initial infection point. (a) Phase durations for hospitals and corresponding community nodes. Phase 1 denotes the beginning of the spread (prevalence lower than 10% of the final prevalence); phase 2 denotes the transition state (prevalence < 99.9% final prevalence). The phase durations correspond to a system-wide prevalence, not to individual facility prevalences. (b) Individual phase ends in healthcare facilities and in corresponding community-nodes. Presented results are averaged-out amid all possible initial infection points. Pathogen spread was initiated by a single infectious patient originating from each facility.

The final states for all simulations are similar, independently of the initially infected healthcare facility (cf. Fig 7). These results suggest that there is a single final state, corresponding to some stationary state of the simulated system. The time to reach the system-wide final state depends on the initial conditions, but differences are very small. The system-wide initial phase lasts about 400-600 days, and then the system-wide transition (second) phase takes up to 7700 days (see Fig 8a).

**Fig 9:**
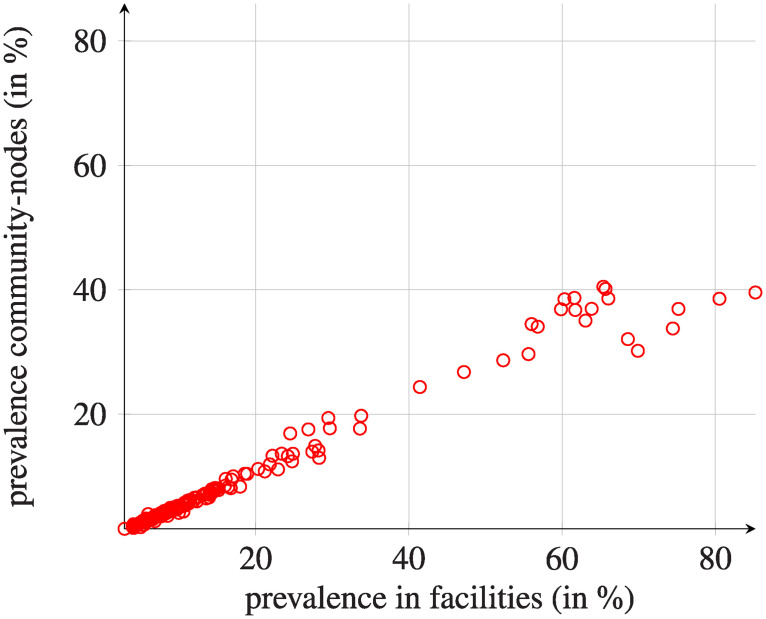
Comparison of the final percentage of infectious individuals in given hospitals/community-nodes. Healthcare facilities are ordered by the average size of hospitals, the smallest first. Transmission was started by a single infectious patient originating from a given facility, and the results are averaged out through all initial facilities. However, differences in the final prevalence between initial originating facilities are negligible.

If we focus on the individual facilities (see Figs 7 and 8b), there is a high heterogeneity in lengths of the studied phases. Some facilities reach their final state within 4000 days; the mean duration of reaching final phase is, however, more than 8000 days. These patterns do not depend on the initial conditions under consideration (except for the initial infection points, where the spread is faster). Moreover, there is no clear association between size of a facility and final prevalence or time to reach it. However, lower final prevalence generally corresponds to a longer transition phase. There is a strong association between the combined length of initial/transition phases for healthcare facilities and their corresponding community-nodes. As expected, the final prevalence for individual community-nodes is generally much lower than the final prevalence of the corresponding facility (Fig 9).

### Influence of SIS parameters on the final prevalence

**Fig 10:**
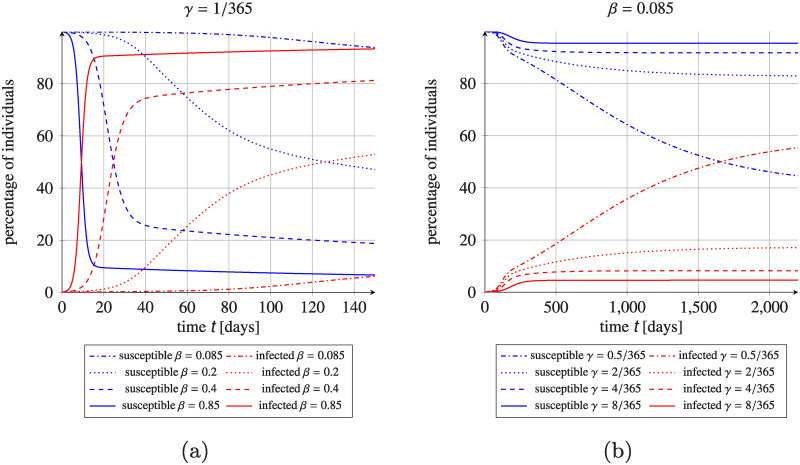
Effect of the change of SIS parameters on MRSA network-wide prevalence. (a) Effect of the change of the transmission rate on the percentage of susceptible (blue) and infectious (red) individuals in the healthcare facilities network. (b) Effect of the change of the recovery rate on the percentage of susceptible (blue) and infectious (red) individuals in the healthcare facility network. Initial fraction of the colonized individuals is 0.1% uniformly distributed in the whole population. Sets of parameters being perturbations of the SIS reference values reported in Table 1 (set type: MRSA) as indicated in legends.

We further investigate the effects of varying transmission rates and recovery rates in the SIS model on the proportion of susceptible and infectious individuals in the healthcare facilities. Fig 10 shows that both parameters, the transmission rate *β* and the recovery rate *γ*, impact the final MRSA network-wide prevalence in line with what would be expected. Larger *β* indicates faster spread of the pathogen while larger *γ* values result in lower prevalence in the whole network at steady state. The system wide effect is smaller for relative changes in *β* than in *γ*.

The length of the initial infection phase seems to be highly dependent on the parameter *β*. This effect is visible in all the presented simulations. When we look closer at the effect of varying recovery rates *γ* (Fig 10), we find a similar behaviour, but the changes are milder.

### Impact of indirect transfers

In order to assess the role of indirect transfers in the pathogen spread, we vary the recovery rates in the community-nodes (*γ*_*c*_). Recovery rates in community-nodes differ from the recovery rates in corresponding hospitals (*γ*_*h*_), as indicated in Table 1 (set type: indirect transfer impact).

**Fig 11:**
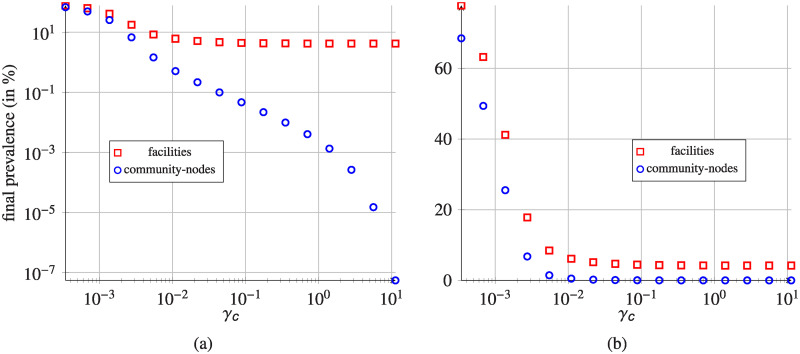
Dependence of MRSA network-wide prevalence in healthcare facilities and corresponding community-nodes (reported for the last day) on different values of *γ*_*c*_ parameter in the community-nodes. Parameter *γ*_*h*_ = 1/365 is kept constant for all the hospitals. Initial fraction of the infectious individuals is 0.1% uniformly distributed in the whole population. (a) Data presented on log-log scale. (b) Data presented on semilog(x) scale.

In Fig 11, we show how the MRSA network-wide prevalence (percentage of infectious individuals) is affected by varying the *γ*_*c*_ parameter in the community-nodes. Starting with ≈ 77% and ≈ 68% prevalence levels in the communities and healthcare facilities respectively, prevalence levels decrease monotonically with increasing *γ*_*c*_. For simulations with *γ*_*c*_ > 128/365 day^−1^ ≈ 0.35 day^−1^, MRSA network prevalence in hospitals stabilizes at the level of about 4.2%. For community-nodes it systematically decreases, falling below 0.1% for *γ*_*c*_ greater than 16/365 day^−1^ ≈ 4.38 × 10^−2^ day^−1^.

**Fig 12:**
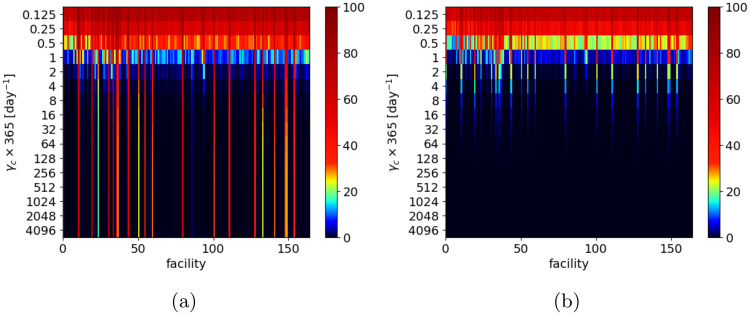
Final MRSA prevalence in the individual healthcare facilities and community-nodes. Final prevalence in the individual healthcare facilities and community-nodes depending on the value of *γ*_*c*_ parameter (vertical axis) for: (a) all considered hospitals, (b) corresponding community nodes. Initial condition for all simulations is the same: 0.1% uniformly-distributed colonized patients. Facilities are sorted by increasing average size.

Fig 12 shows how the final prevalence in the individual healthcare facilities and community-nodes varies with changing *γ*_*c*_ parameter. There is a small fraction of facilities, where the prevalence is very high, independent of the community recovery rate (*γ*_*c*_). We observe a similar effect for communities, but for sufficiently high *γ*_*c*_, all communities have a very low prevalence.

**Fig 13:**
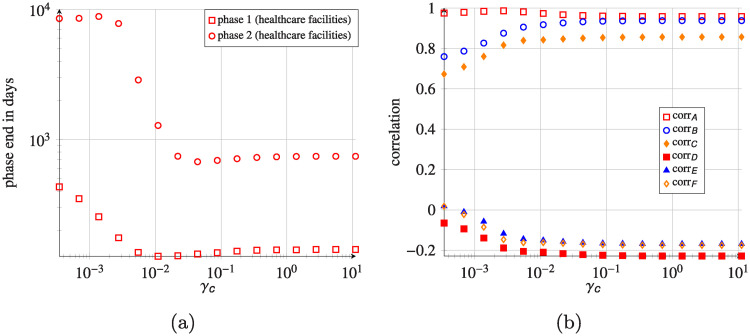
System-wide phase ends in healthcare facilities and correlation coefficients. (a) System-wide phase ends in healthcare facilities for different *γ*_*c*_. (b) Dependence of the correlation coefficients on *γ*_*c*_ value (semi-log scale), corr_*A*_ is the correlation coefficient between final hospital prevalences and corresponding communities prevalences; corr_*B*_—correlation coefficient between final hospital prevalences and average length of stay in hospitals; corr_*C*_—correlation coefficient between final community prevalences and average length of stay in corresponding hospitals; corr_*D*_—final prevalence in hospitals and hospital in-degrees; corr_*E*_—final prevalence in hospitals and hospital out-degrees; corr_*F*_—final prevalence in community nodes and community out-degrees. Initial condition for all simulations is the same 0.1% uniformly-distributed colonized patients.

Time needed to reach the final prevalence state is another important feature affected by the inclusion of indirect transfers. In Fig 13a, we looked at the time needed to reach the end of phase 1 (prevalence lower than 10% of the final prevalence) and phase 2 (i.e. prevalence < 99.9% final prevalence). Interestingly, the time to reach the end of phase 1 shows a monotonous decrease for small *γ*_*c*_, while it is not the case for the end of phase 2.

We further computed correlation coefficients between the final prevalence in the hospitals and community nodes (corr_*A*_), the final prevalence in the hospitals and the average length of stay in hospitals (corr_*B*_) and final prevalence in communities and the average length of stay in hospitals (corr_*C*_), for different values of *γ*_*c*_. Fig 13b shows that all mentioned correlation coefficients are strongly positive for all the considered *γ*_*c*_ values. For small *γ*_*c*_, the strongest correlation exists between the final prevalence in the hospitals and corresponding community-nodes. However, it slightly decreases with increasing *γ*_*c*_, while at the same time, correlations between final prevalence in the hospitals (or in communities) and the average length of stay in hospitals increase. For a sufficiently large community recovery rate (*γ*_*c*_ > 0.1 day^−1^), when pathogen transfer via community nodes is minor or negligible, all the correlations stabilise at certain levels and become independent of *γ*_*c*_. The investigation of correlations between the final prevalence in the hospitals (or the community-nodes) and in- or out-degree of hospital nodes (or out-degree of community-nodes) brought no statistically significant results.

### Influence of the length of stay on prevalence

**Fig 14:**
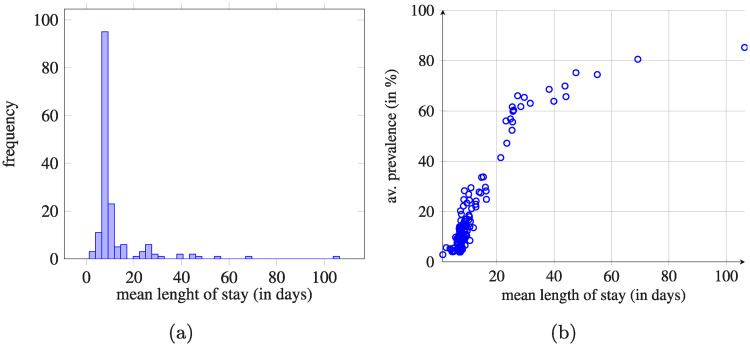
Distribution of length of stay and the dependence between the average length of stay and average prevalence. (a) Distribution of length of stay (in days) for all the considered hospitals (164 units). (b) Average length of stay vs. average prevalence calculated for each hospital.

We additionally looked at the distribution of average length of stays for the considered hospitals (see Fig 14a). In most cases, it was between 7 and 9 days. Moreover, Fig 14b shows the correlation between the average length of stay and the prevalence for all the considered hospitals. The prevalence for each hospital was calculated based on the simulations presented in Sec. Impact of the initial infection point by taking the average from the simulations where the origin of the colonization was changed (164 simulations in total). The obtained relationship is non-linear, but the deviation from linearity is observed for few facilities with longest average length of stay.

## Conclusion and discussion

In this work, we showed how to derive a healthcare network from an anonymized admission/discharge data based on dataset provided by AOK Lower Saxony, Federal Republic of Germany. For every healthcare facility in the regional healthcare network, we added a corresponding community-node containing patients waiting for re-admission to the same or a different healthcare facility. We further simulated the transmission dynamics of MRSA as an example for multidrug resistant bacteria in the proposed network model of patient traffic, taking into account indirect patient transfers. Due to the characteristics of pathogens for which transmission occurs mostly in hospitals, patient exchange between facilities is a main driver of their spread. Our analysis of the AOK Lower Saxony anonymized dataset for the years 2008–2015 shows that patient transfers are well balanced between the facilities. Thus, we can use a deterministic model, and in particular a Markov chain, to describe the patient movements within the network. Nevertheless, the derived probability matrix defining the Markov process is not symmetric, indicating that the directed graphs should be used instead of the indirected ones. Although we propose a simplified deterministic transfer model, the stationary probability distribution (eigenvector corresponding to eigenvalue equal to 1) are close to values estimated directly from data, underlining the correctness of the proposed approach.

We found that only 6% of transfers in our underlying dataset are direct transfers; the remaining 94% include some time in the community before readmission. From these transfers, we deduced a Markov process transfer probability matrix, which in combination with a SIS intra-hospital model determines the transmission dynamics of MRSA within the healthcare network. We found that a typical spread pattern in such a network consists of three phases. In the first phase (*initial*), the percentage of infectious patients is close to 0 and the length of this phase is strongly associated with the transmission rate *β*. In the second phase (*transient*), the prevalence rapidly increases, and then it reaches a stable level, which is the final third phase (*stable*). Length of these phases and final prevalence depends on *β* and on the recovery rate *γ*. In addition, we observed that the stable-state colonization rate of the individual facilities is not uniform (despite uniform *β* and *γ*). Mean length of stay is one of the major determinants of prevalence at steady state (understood as the state after sufficiently long time, when the changes are not pronounced anymore) with the longer stays being associated with higher prevalence. The node position in the network may also play an important role; however, our analysis of node degrees shows no statistically significant correlation between in-degrees (or out-degrees) and the prevalence in hospital nodes or corresponding community nodes. Additionally, we observe that the final prevalence in the healthcare facilities is independent of the facility, from which the pathogen spread was initiated in; however, the initial dynamics may differ slightly.

In the main part of our analysis, we found that taking indirect transfers into account has a relevant effect on the final prevalence in the healthcare system. We show that spread of the pathogen by direct transfers only leads to 4.2% final system wide prevalence, while including indirect transfers leads to a prevalence increase to almost 18%. While the relationship can be different for other pathogens, this indicates that indirect transfers are as important or even more important as direct transfers and cannot be ignored. Limiting the simulation to the healthcare system, and ignoring the indirect transfers and the community, may result in misleading results. Not only the role of transfers is misrepresented, but there are also differences in the transmission dynamics when indirect transfers are omitted. For high *γ*_*c*_ (corresponding to transmission of MRSA via the direct transfers only) the system reaches the stable state in 2–3 years, while with indirect transfers it needs about 20 years to reach almost 18% prevalence.

Moreover, using the proposed model, we also found a non-linear concave functional dependence of the prevalence in the hospitals on the average length of stay in these facilities. This can be a useful tool for the estimation of prevalence in given hospitals, knowing the average length of patient stays of the considered facility. This dependence is based strongly on the network structure, and in the future we will address this issue by studying different hospital networks.

One example of emerging multi-resistant pathogens, which are more complex to model than MRSA, are multi-resistant gram-negative bacteria, especially *Enterobacteriacaeae*. In [22], the prospective surveillance data from 13 European intensive care units were used to estimate transmission rates of *Escherichia coli* and *E. coli Enterobacteriaceae*. This group of pathogens shares resistance genes, but has very different transmission characteristics, transmission ways and clearance rates, making the correct choice of time frames for indirect transfers more difficult [22].

Infection control by adjustment of the network structure (as e.g. proposed by Donker et al. in [9]), provides another level of complexity which can be considered in combination with classic infection control measures focusing on individual healthcare facilities. In [4], Lee et al. have shown that the distribution of infection control measures based on network characteristics can improve the effectiveness of infection control measures and save resources at the same time. However, our analysis showed that the decrease of the transferability of the pathogen via the community does not significantly influence the correlation between the final MRSA network-wide prevalence (in the facilities and corresponding communities) and average lengths of stays in hospitals.

To conclude, we showed that indirect transfers are important in the spread of pathogens in a healthcare network. Dependent on the type of pathogen studied and its clearance rate, network flows representing indirect transfers within a pre-defined time frame need to be taken into account to understand the spread of pathogen due to transfers in a healthcare network, and the effectiveness of potential infection control measures. The study presented in this paper was performed for a single federal state in Germany. Provided that there are available data, the same may be done for additional regions or a group of countries. One further challenge will be to determine patient transfers between administrative regions. Our model can be further expanded by taking into account infection control measures. This requires more detailed modelling at the intra-hospital level. Screening, isolation of colonized patients and introduction of effective treatment may be simulated for example by decreasing *β*. Also, additional stratification of risk may be added by similar means. The analysis of the influence of hospital heterogeneity, expressed e.g. by different transmission rates, should be also addressed in the future, provided access to relevant epidemiological data.

## Supporting information

S1 Appendix Lemma. Stochastic-regularity of probability transfer.
(PDF)

S2 Appendix Transfer classification algorithm. (PDF)

S3 Appendix On the impact of transmission rate in community. (PDF)

S4 Appendix Examples of length of stay distributions. (PDF)

S5 Appendix Phase durations. (PDF)

S1 Data Numerical data. Numerical data for Figs 3, 4, 6, 7a and 7b.
(ODS)

S2 Data Numerical data. Numerical data for Figs 7c, 7d and 8–14.
(ODS)
