# Supplementary material for: Modelling pathogen spread in a healthcare network: Indirect patient movements
Source: PLoS Comput Biol. 2020 Nov 30;16(11):e1008442. doi: 10.1371/journal.pcbi.1008442 (PMC7728397; doi:10.1371/journal.pcbi.1008442)
Supplement: S1 Appendix — Stochastic-regularity of probability transfer. (PDF) [file pcbi.1008442.s001.pdf]

S1 Appendix: Lemma

Lemma 1 *Assume that* $A = [A_{ij}]_{i,j=1}^{k}$ *is a* $k \times k$ *dimensional real matrix such that*

1. $\forall i, j \in \{1, \ldots, k\}$, $i \neq j$, $A_{ij} \geq 0$,
2. $\forall j \in \{1, \ldots, k\}\ A_{jj} > 0$,
3. $\forall i \in \{1, \ldots, k\}\ \sum_{j=1}^{k} A_{ij} = 1$,
4. $\forall i, j \in \{1, \ldots, k\}\ \exists\, i_0 = i, i_1, \ldots, i_{n-1}, i_n = j$ *such that* $\forall m \in \{1, \ldots, n\} \quad A_{i_{m-1} i_m} > 0$.

Then A is a stochastic regular matrix.

The proof of the following lemma can be find in [14].

This lemma may be applied to hospital transfer probability matrices of this paper. Clearly, Assumption 1 of Lemma 1 is satisfied based on definition (4), as all the elements are non-negative. Assumption 2 is more subtle, as in general A_{jj} can be equal to 0. It is, however, rather unlikely, as due to (4) it would imply that no patients would ever stay in the healthcare facility overnight. Assumption 3 is a direct consequence of definition (5). Assumption 4 means that for every two facilities, there is some (potential) transfer path between them. Actually, it is not necessary that any patient follows this whole path, but there must be some patient transfer for every component. Thus, the transfer path must exist between every two facilities, in both directions.
