# Supplementary material for: Modelling pathogen spread in a healthcare network: Indirect patient movements
Source: PLoS Comput Biol. 2020 Nov 30;16(11):e1008442. doi: 10.1371/journal.pcbi.1008442 (PMC7728397; doi:10.1371/journal.pcbi.1008442)
Supplement: S2 Appendix — (PDF) [file pcbi.1008442.s002.pdf]

S2 Appendix: Transfer classification algorithm

To classify patient transfers properly, we propose a heuristic algorithm, which detects and classifies transfers from the claims database in a general manner. Please note that we use in description of the algorithm: i to denote days, l for facilities, p for patients and symbols A_i, d, D, H_i, L_i, P_i defined below. This notation is local, i.e. it will be used in description of this algorithm, however some of these symbols are used elsewhere for different notions.

Our algorithm operates at the basis of individual patients and works as follows. Let H denote a set of healthcare facilities. Let us take a patient and the admission/discharge table related to his/her hospital stays. Then by $D := \{0, 1, \ldots, d\}$ we denote a set of days on which this patient stays in any healthcare facility. We assume that the order of the indices D corresponds to the order of days, but it is possible that these days are not consecutive, i.e. days i and $i + 1$ may actually be separated by some period of time.

Then let $H_i \subset H$ be a set of healthcare facilities in which the patient formally stays at day i, i.e. there is at least one entry in the considered admission/discharge dataset, which corresponds to this patient while the day i is between the admission date and discharge date of this entry (both inclusive). Moreover, we define $H_{-1} := \emptyset$ and a set of facilities to which the patient was recently admitted as $A_i := H_i \backslash H_{i-1}$, while a set of facilities which the patient recently left is denoted by $L_i := H_{i-1} \backslash H_i$.

H_i can have more than one element when entries overlap and patients are formally assigned to multiple facilities at the same day. To track such situations, we also define sets $P_i \subset H$, which correspond to facilities in which we think the patient is present on day i. These sets will be determined by the proposed heuristic.

If $i - 1$ and i are consecutive days, we check every new facility $a \in A_i$. Then for every $p \in P_{i-1}$ we deduce a transfer from p to a.

Then for any facility $l \in L_i \cap P_{i-1}$ which the patient formally leaves on day i and in which he/she was present on day $i - 1$, and for any facility $h \in H_i$ in which he/she formally is on day i, we deduce a transfer from l to h. We take $h \in H_i$ instead of for example $h \in P_{i-1}$. We must account for the case, when a patient has a temporary transfer to another facility. When the new stay finishes, the patient returns to the original facility, in which he/she is now considered as not present (the facility is not in P_{i-1}), but formally he/she is registered there (it is in H_i).

We determine P_i as follows:

- If there is no new admission or discharge ($A_i = L_i = \emptyset$), then nothing changes ($P_i := P_{i-1}$).
- If there are new admissions ($A_i \neq \emptyset, L_i = \emptyset$), then we deduce that the patient is transferred to these new locations and $P_i := A_i$.
- If there are discharges ($L_i \neq \emptyset$), then any facility left is removed from P_i; all current facilities are added to this set, as we assume that the patients are returning to the original facilities. So, depending on whether there were new admissions ($A_i \neq \emptyset$) or not ($A_i = \emptyset$), we define $P_i = (A_i \backslash L_i) \cup H_i$ or $P_i = (P_{i-1} \backslash L_i) \cup H_i$.

On the other hand, if i and $i + 1$ are not consecutive days, i.e if there is at least one day in between, then for every $p \in P_{i-1}$ and for every $a \in A_i$ we deduce a transfer from p to a and we set $P_i := A_i$. Note that it is possible that $P_{i-1} \cap A_i \neq \emptyset$, thus we can deduce indirect auto-transfers also. Since we need some initial value, we define $P_{-1} := \emptyset$ and we assume that -1 and 0 are not consecutive days.

This procedure is executed for each patient from the dataset; afterwards transfers are accumulated.
