# Supplementary material for: Modelling pathogen spread in a healthcare network: Indirect patient movements
Source: PLoS Comput Biol. 2020 Nov 30;16(11):e1008442. doi: 10.1371/journal.pcbi.1008442 (PMC7728397; doi:10.1371/journal.pcbi.1008442)
Supplement: S3 Appendix — (PDF) [file pcbi.1008442.s003.pdf]

S3 Appendix: On the impact of transmission rate in community

In the proposed model, we treat the communities as artificial containers of former patients of corresponding hospitals waiting for a new admission in the community. It is important to note that coexisting in the same community on the one hand does not necessary imply geographical closeness of people, and on the other hand, it is likely that some of these communities overlap geographically to a great extent. Thus, it is not feasible to treat these communities as separate entities and to model pathogen spread within them by similar means as in healthcare facilities.

However, we may consider an idealized case, where we assume that there is spread of infections in the community nodes. In such a case, we would like to demonstrate that the transmission rate in communities has to be relatively high to have sufficient impact on the system to actually make a qualitative difference. We made a preliminary simulation, where we set the transmission parameter β_c in the communities to a positive value. The effect of this additional transmission on the prevalence in healthcare facilities and in community is presented in Figs. A and B.

For low transmission in the community, the overall prevalence is almost the same as for no-transmission case. Moreover, the prevalence pattern and time scale are similar. Only for community transmission parameter higher than 1% of hospital transmission parameter the differences start to be substantial.

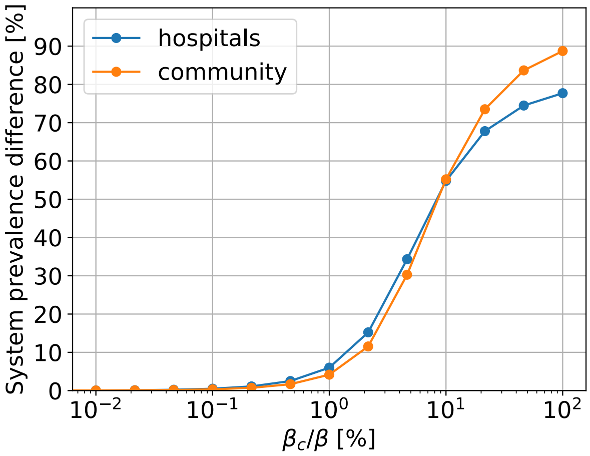

Figure A: Visualisation of the difference between system prevalence in healthcare facilities and in community for simulations with non-zero transmission parameter in the community β_c.

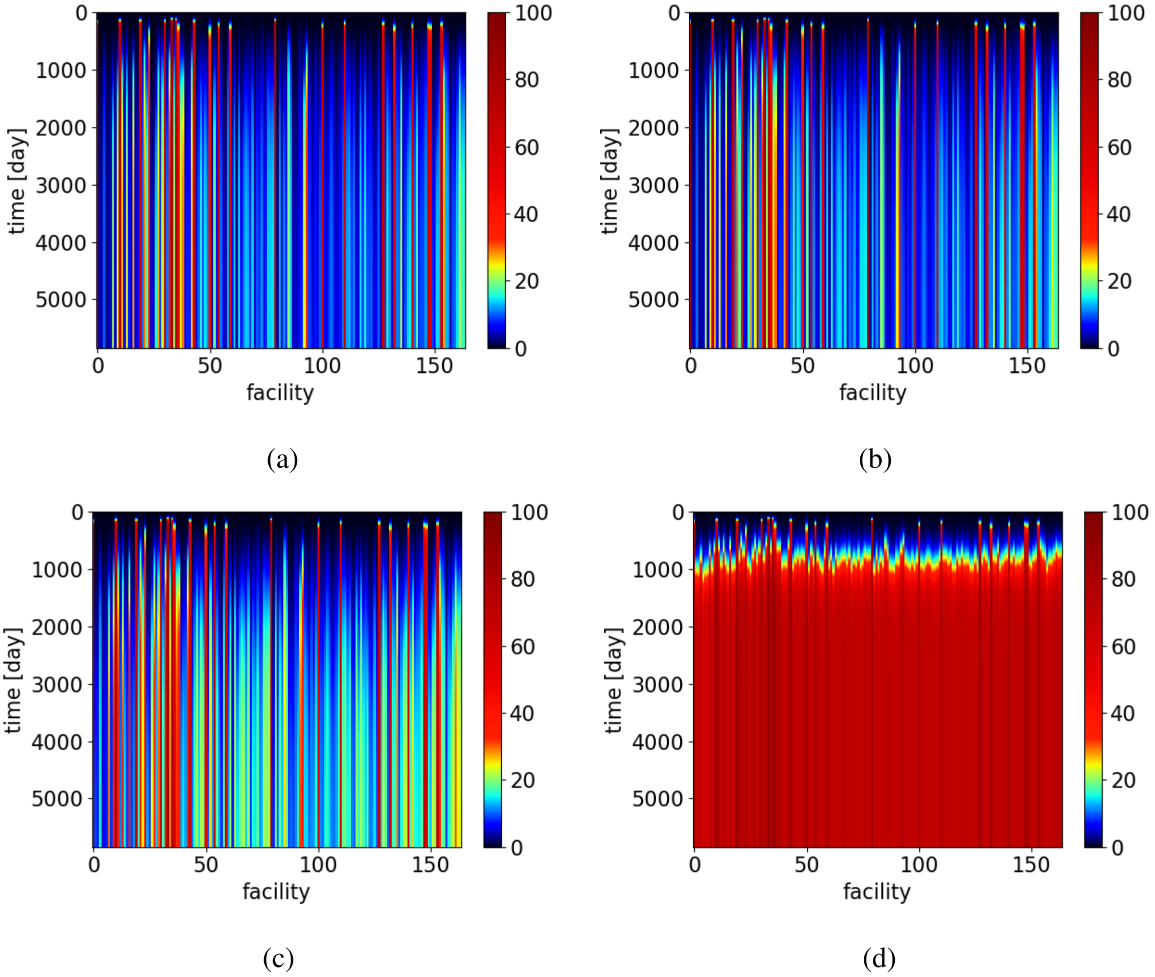

Figure B: Prevalence vs time for healthcare facilities, sorted by size (smallest first), for the following β_c/β ratio: (a) 0 (original case), (b) 0.1%, (c) 1%, (d) 10%.
