# Supplementary material for: Modelling pathogen spread in a healthcare network: Indirect patient movements
Source: PLoS Comput Biol. 2020 Nov 30;16(11):e1008442. doi: 10.1371/journal.pcbi.1008442 (PMC7728397; doi:10.1371/journal.pcbi.1008442)
Supplement: S4 Appendix — (PDF) [file pcbi.1008442.s004.pdf]

S4 Appendix: Examples of length of stay distributions

In Fig. A we present few examples of distributions of length of stay in selected hospitals. While they roughly resemble exponential decay, there are some exceptions. In particular we see that shorter stays are more common.

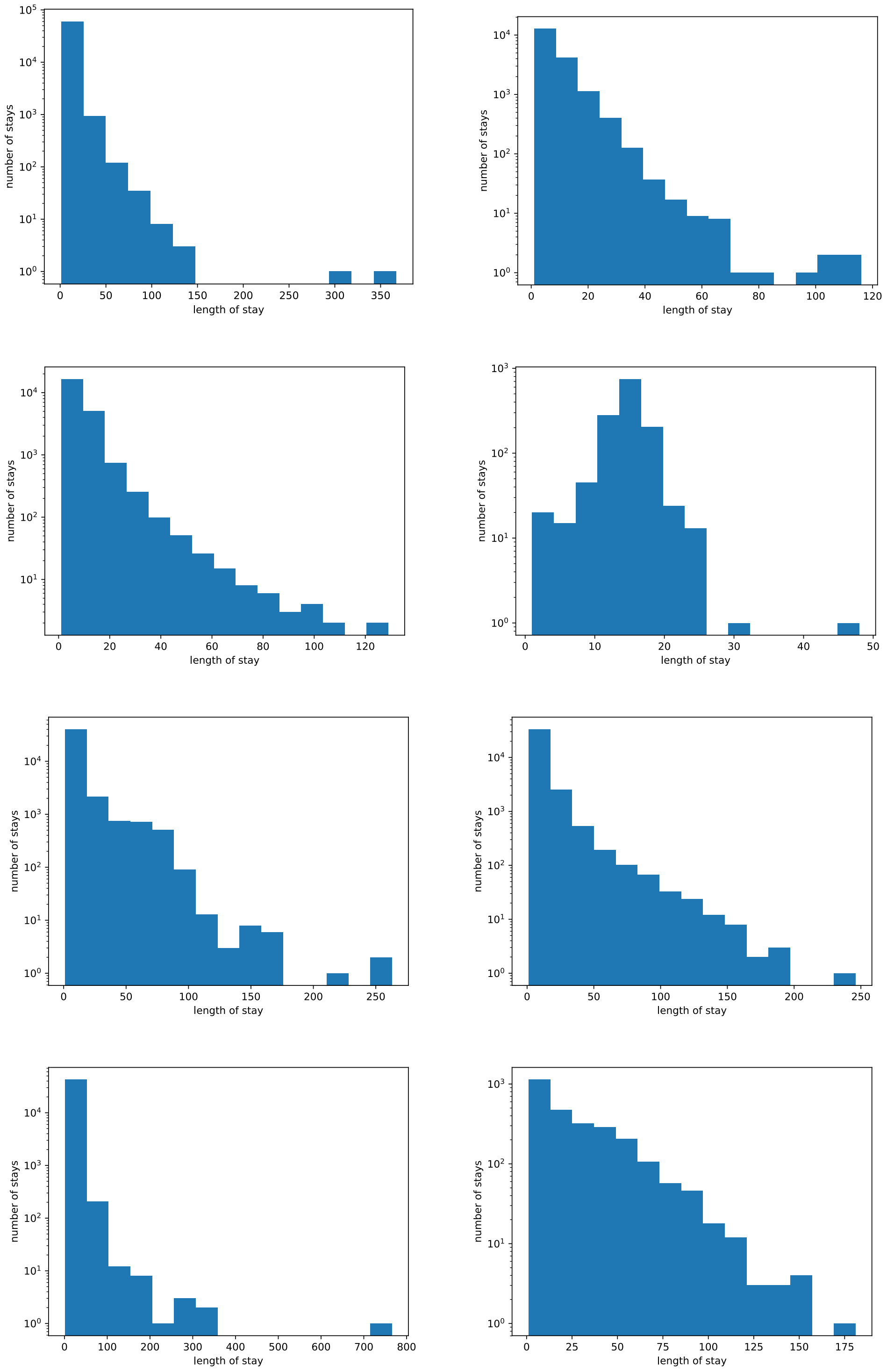

Figure A: Some examples of distributions of length of stay in different hospitals taken form the data. Number of stays is given in logarithmic scale.
