# Supplementary material for: Modelling pathogen spread in a healthcare network: Indirect patient movements
Source: PLoS Comput Biol. 2020 Nov 30;16(11):e1008442. doi: 10.1371/journal.pcbi.1008442 (PMC7728397; doi:10.1371/journal.pcbi.1008442)
Supplement: S5 Appendix — (PDF) [file pcbi.1008442.s005.pdf]

S5 Appendix: Phase durations

For the purpose of our paper, we defined two phases of the infection spread. The initial phase (which duration is defined by time to reach the prevalence of 10% of the final prevalence), the (second) transition phase (which duration is defined by difference between time to reach the level of 99.9% of final prevalence and the end of the initial phase), cf. Fig. A. Clearly, the transition points were chosen arbitrary but is such a way that we were able to describe and compare the results for different facilities or different simulations.

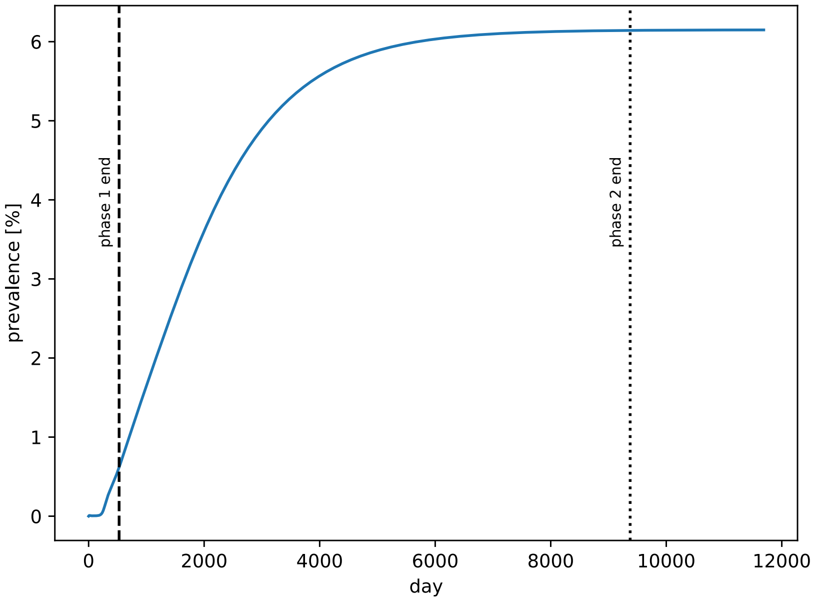

Figure A: Representation of the infection phases in case of the plot of the dependence of the prevalence in time for randomly chosen hospital and experiment.
